# A multidisciplinary approach to managing severe gummy smile using 3D simulation and digital surgical guide: a case report

**DOI:** 10.1093/jscr/rjae483

**Published:** 2024-08-06

**Authors:** Hoang Viet, Dang Thi Nhu Thao, Tran Hong Phuoc, Nguyen Quang Tien

**Affiliations:** Department of Orthodontics and Pedodontics, Faculty of Dentistry, Van Lang University, 69/68 Dang Thuy Tram, Ward 13, Binh Thanh District, Ho Chi Minh City, 70000, Vietnam; Department of Orthodontics and Pedodontics, Faculty of Dentistry, Van Lang University, 69/68 Dang Thuy Tram, Ward 13, Binh Thanh District, Ho Chi Minh City, 70000, Vietnam; Department of Oral Surgery, Sai Gon Dental Private Hospital, 1256-1258 Vo Van Kiet, Ward 10, District 5, Ho Chi Minh City, 70000, Vietnam; Department of Orthodontics, Sai Gon Dental Private Hospital, 1256-1258 Vo Van Kiet, Ward 10, District 5, Ho Chi Minh City, 70000, Vietnam

**Keywords:** gummy smile, gingivoplasty, digital, orthodontics, multidisciplinary, surgical guide, 3D printing

## Abstract

A smile that reveals >4 mm of gum tissue is called a gummy smile (GS), offering negative impacts on people’s self-confidence and aesthetic appearance. The treatment for GS should be planned according to underlying causes such as altered passive eruption of teeth, dentoalveolar extrusion, vertical maxillary excess, and short or hyperactive lip muscles. In this case report, a patient with severe GS received orthodontic and gingivoplasty treatment, aided by digital tools such as 3D simulation, smile design, and 3D printed guides. The treatment yielded remarkable and satisfactory results, without the need for extensive surgery. Our findings suggest that gingivoplasty is a minimally invasive, time- and cost-effective alternative to more extensive procedures for correcting severe gum recession.

## Introduction

Excessive gingival display or ‘gummy smile’ (GS) is gingival display of >4 mm of gingiva, often considered an undesired smile. Factors that contribute to the GS include altered passive eruption, lip length, lip hypermobility, incisal wear/crown length, vertical maxillary excess, and gingival hyperplasia [1]. Depending on these problems, there are various treatment options such as gingivectomy, orthodontics, botulinum toxin injections, plastic reconstructive surgery, periodontal surgery, or orthognathic surgery.

The purpose of this article is to review the aetiology, diagnosis, and non-surgical approaches in treating the GS. In this case report, we would like to demonstrate the advantages of digital applications in orthodontic and periodontic treatment. The use of 3D simulation, smile design, and 3D printing digital guide makes the treatment process more accurate and predictable, while also simplifying the clinical procedure [1–3].

## Case presentation

A 16-year-old patient came to our clinic with several chief complaints including the gaps between the teeth, GS, and protrusion.

**Figure 1:**
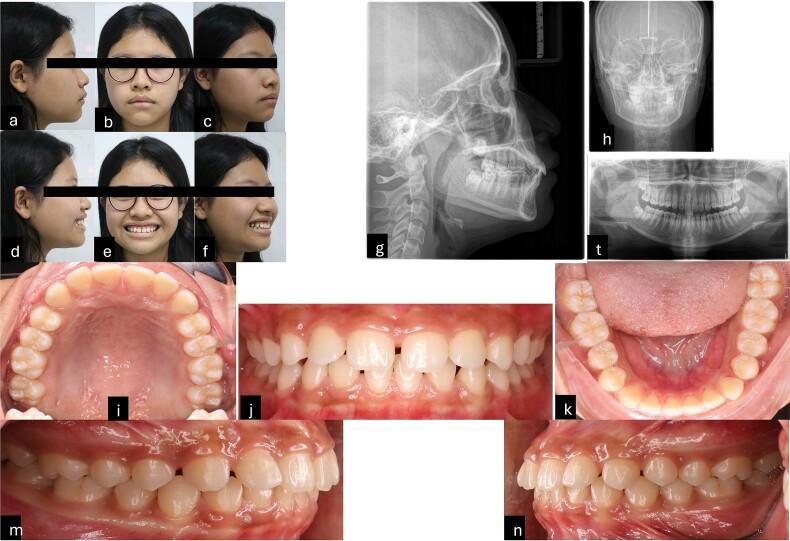
Pre-treatment records. (a–f) Extra oral pictures; (g–t) pre-treatment radiographies; (l–n) intraoral pictures.

Intraorally, the patient is skeletal Class II with Class I molar, deep curve spee, anterior spacing upper and lower, abnormal position of the tongue and tongue posture, and short teeth were also noted. Extraorally, the patient had convex profile, proclined the upper incisors and GS but the upper lip of the patient was asymmetrical during smiling. The pre-treatment radiographies showed proclined upper incisors, deep curve spee, spacing, and four impacted wisdom teeth (Fig. 1).

The patient’s smile revealed 5 mm of gums with clinical manifestations of short and square tooth crowns. The ratio between the length and width of the tooth crown clinically was smaller than on the radiographies. Based on cone beam computed tomography, the alveolar crest of the maxillary anterior teeth was determined to be ~1 mm away from the cementoenamel junction (CEJ). However, a direct assessment of the relationship between the alveolar crest and CEJ using the flap method would be more accurate than surveying on radiographies. Based on the evidence, it was determined that the patient had a GS due to passive teething type 1a, which the osseous crest levels were found to be ~1.5 to 2 mm from the CEJ [4] and hyper mobile lip [5]. In addition, the patient has deep bite with an overbite of 4 mm, the occlusal plane difference between anterior and posterior. Therefore, the cause of a GS is a combination of altered passive eruption and overeruption of the maxillary incisors.

### Clinical findings

Soft tissue:

- Convex profile
- Acute nasolabial angle
- Strain on circumoral muscle when closing mouth (no TMJ symptoms)

Lip position assessment:

- Upper and lower lips protrusive
- Both lips anterior to E-line
- Hypermobile lip
- Smile: GS 5 mm
- Buccal corridors: normal

Dental:

- Molar relationship: Class I
- Canine relationship: Class I
- Archform:
  - Upper: normal
  - Lower: normal
- Upper arch:
  - 4 mm spacing
  - Extruded anterior teeth
- Lower arch:
  - 2 mm spacing
  - Deep curve of spee
- Bolton discrepancy:
  - Anterior: 3–3: 78% (normal)
  - Posterior: 91% (normal)
- Midline: upper and lower coincident with facial midline

Skeletal:

- Skeletal jaw relationship: Class II (ANB 4.7°)
- Maxilla and mandible: protruded position (SNA 88.5°, SNB 83.8°)
- Lower facial height: normal (FMA 22.1°)
- Incisor angulation
  - Upper: normal (U1-SN 109.7°)
  - Lower: proclined (L1-MP 98.5°)

#### Treatment objectives

- Eliminate the bad habit, anterior spacing, GS, and reposition the tongue.
- Achieving satisfactory smile aesthetics, stable occlusion, and masticatory function in a long term.

#### Potential alternative treatments

We presented several treatment options, including the choice between the current treatment plan and treatment combined with clear aligner and gingivoplasty or with botulinum toxin after treatment. However, the patient’s parents expressed concerns about compliance of her daughter. Consequently, she opted for the treatment with fixed appliance and gingivoplasty. In the case of passive erupted teeth and hyper mobile lips, botulinum toxin can be employed; however, she refused to use it.

### Treatment progress

**Figure 2:**
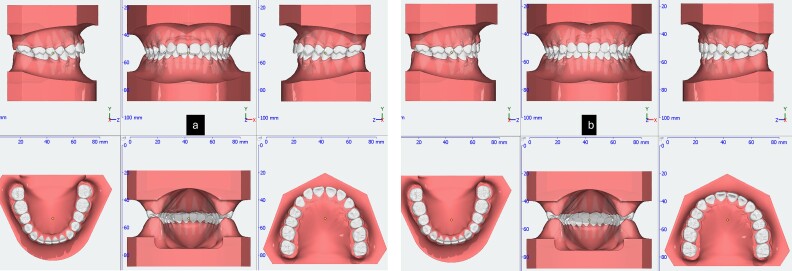
3D simulation of pre-treatment (a) and post-treatment (b).

**Figure 3:**
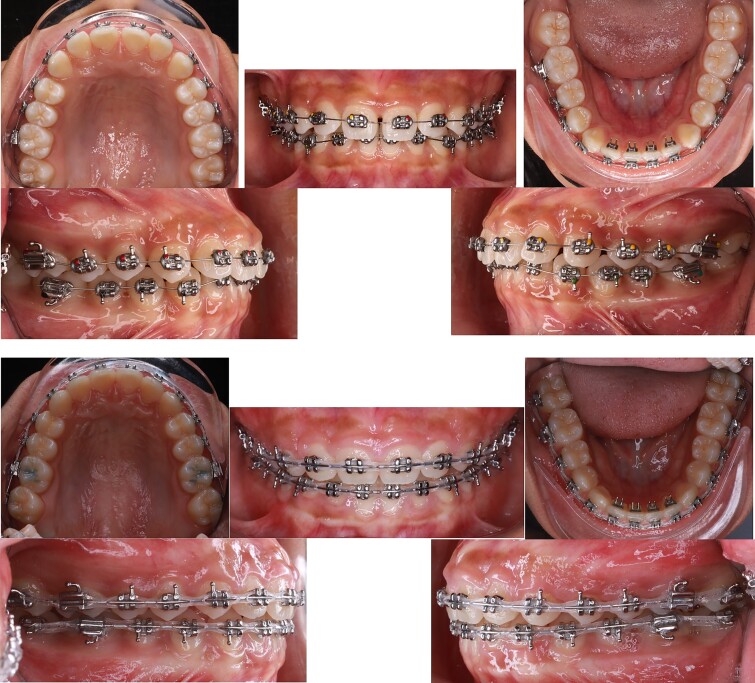
Tongue spurs bonding, alignment, leveling and space closure phase of the treatment.

During the initial stage, orthodontic treatment was utilized to close the gaps between the teeth and move the upper and lower front teeth back using a fixed appliance, reverse curve wire, and power chain to achieve control tipping movement and improve her convex profile [6]. Tongue spurs were attached to keep the tongue in the correct position and prevent gaps from forming between the teeth. At the same time, a wire was used to intrude the upper front teeth during orthodontic treatment. The 3D simulation [7] before the treatment was done for consulting with patient and patient’s family (Fig. 2). The following procedures were performed: a fixed appliance was attached to both the upper and lower arches. Over a period of 4 months, alignment and levelling were done using a Niti wire, followed using a stainless-steel wire to close all the spaces using a sliding mechanic with reverse curve stainless steel wire bending on 0.017 × 0.025 SS and power chain. In the final 3 months, the detailing and finishing stage were completed to achieve functional occlusion (Fig. 3).

**Figure 4:**
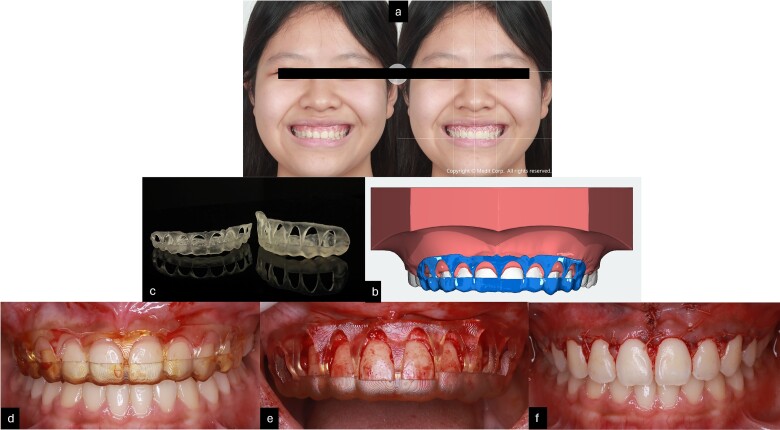
(a) Smile design before gingivoplasty treatment; (b) 3D printing gingivoplasty guide; (c) 3D guide design; (d,e) gingivoplasty treatment with guide; (f) post-treatment.

The crown-root ratio of the upper front teeth can be adjusted by removing enough bone while maintaining the biologic width. This involves two procedures: osteotomy, which entails removing enough bone while maintaining the biologic width, and osteoplasty, which involves curving the alveolar crests, creating lateral grooves, and thinning the bone border. To make the surgery simpler and more controlled, 2D smile design [8] on smile picture a 3D simulation, and an 3D guide design [9] were used to create a surgical guide for the gingivoplasty procedure. The treatment was performed by periodontist with laser for gingivectomy and handpiece for gingivoplasty (Fig. 4).

### Treatment results

**Figure 5:**
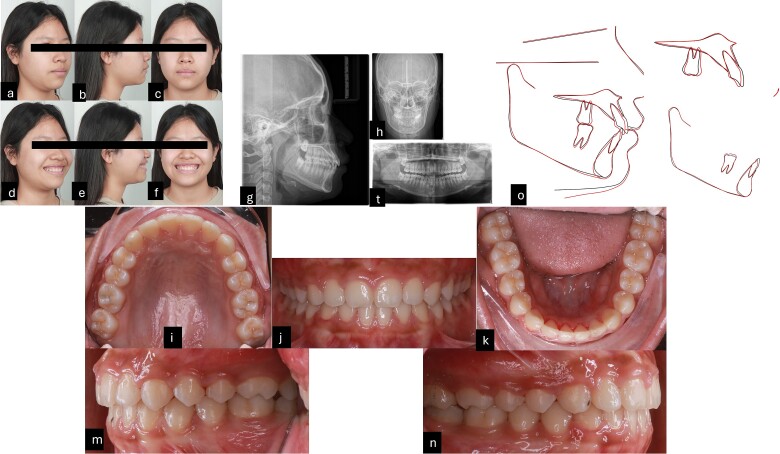
Post-treatment records. (a–f) Extra oral pictures post-treatment; (g–t) post-treatment radiographies; o: superimposition before and after treatment; (i–n) intraoral pictures post-treatment.

**Figure 6:**
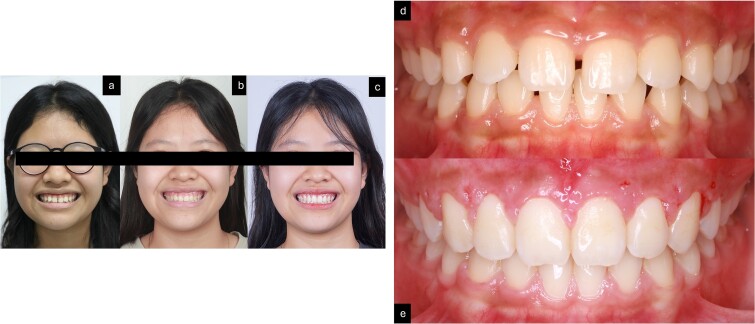
Smile of the patient. (a) Pre-treatment; (b) after orthodontic treatment; (c) after gingivoplasty treatment; (d) before treatment; (e) after 2 weeks of gingivoplasty treatment.

**Figure 7:**
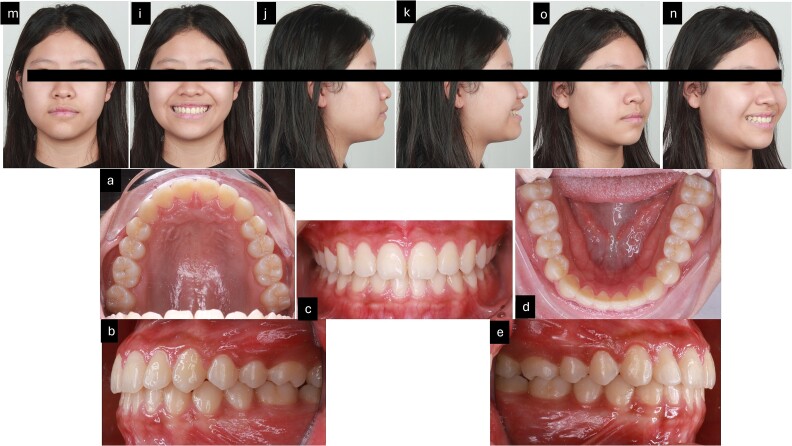
3 months follow-up after treatment. (m–n) Extra oral pictures; (a–e) intraoral pictures.

After 16 months of orthodontic treatment, all treatment objectives were successfully accomplished. This resulted in a well-aligned dentition with all spaces closed, leading to enhanced facial aesthetics, and retracted upper incisors. The overbite and overjet were within normal ranges, achieving a Class I occlusion. There was also a visibly reduced GS after treatment. The superimposition and cephalometric analysis table before and after treatment showed significant improvements in profile and incisor inclination, the clear aligner removable appliance was used for retention (Figs 5 and 6, and Table 1). After 2 weeks of periodontal surgery, the patient’s gingival line has healed exceptionally well. It now wraps around the tooth roots in a scalloped shape, with no black triangles or exposed tooth roots. The surgery also fixed the patient’s GS, resulting in an ideal gingival exposure. As a result, the crowns of the teeth are more visible and stable up to 3 months (Fig. 7).

**Table 1:** Cephalometric analysis pre- and post-treatment.

**Measurement**	**Norm**	**Pre-treatment**	**Post-treatment**	
SNA (°)	81.1 ± 3.7	88.5	87.6	**Skeletal**
SNB (°)	79.2 ± 3.8	83.8	84.2	
ANB (°)	2.5 ± 1.8	4.7	3.4	
FMA (°)	25.0 ± 4.0	22.1	24.3	
U1 – SN (°)	105.3 ± 6.6	109.7	102.8	**Dental**
U1 - NA (mm)	4.0 ± 3.0	4.3	4.6	
U1-NA (°)	22.0 ± 5.0	21.2	15.2	
U1 - L1 (°)	128.0 ± 5.3	120.7	133.7	
L1 – NB (mm)	4.0 ± 2.0	7.4	6.3	
L1-NB (°)	25.0 ± 5.0	33.4	27.7	
IMPA (°)	90.0 ± 3.5	98.5	90.6	
UL – E line (mm)	0 ± 2	2.5	0.9	**Soft tissue**
LL – E line (mm)	0 ± 2	0.7	0	

ANB: A point, nasion, B point, FMA: Frankfort mandibular plane angle, IMPA: incisor mandibular plane angle, L1: lower central incisor, LL: lower lip, MP: mandibular plane, NA: nasion point A; NB: nasion point B, SNA: sella nasion point A, SNB: sella nasion point B, U1: upper central incisor, UL: upper lip. E-line: Ricketts.

## Discussion

In addressing spacing, protrusion, and GSs, the combined approach of orthodontic and periodontic therapy is considered a common option. This comprehensive treatment not only targets visible issues but also tackles their underlying causes. Additionally, it offers a low recurrence rate, compared to alternative cosmetic solutions such as fillings, veneers, or porcelain restorations. However, it requires a longer duration to achieve its desired results.

To plan the treatment for a GS, it is crucial to assess the initial clinical condition thoroughly. This includes evaluating the relationship between the gingival margin, alveolar crest, CEJ, and the crown-root ratio. Coslet and others [10] classified APE into two case types, based on the gingival and osseous relationships. Type 1 presents a noticeably wider band of keratinized tissue, and Type 2 exhibits a smaller band of keratinized tissue falling within normal limits. Types 1 and 2 each have subcategories, A and B. In the A subgroup, the osseous crest is located 1.5 to 2 mm below the CEJ (normal), while in the B subgroup, the osseous crest is found directly adjacent to the CEJ. These assessments will help determine the appropriate treatment option, which could involve gingivectomy with or without bone resection or other treatments. Combining orthodontic and periodontic treatment as multidisciplinary treatment [11, 12] using digital applications can speed up the treatment process, resulting in satisfactory aesthetic outcomes for patients. Using 3D simulation prior to orthodontic treatment allows for discussion with parents and patients about the treatment plan, as well as prediction of the mechanics involved. Currently, the 3D digital workflow assists clinicians in performing precise work more efficiently, reducing chair time compared to conventional treatment management [13–15]. Additionally, with smile design and a 3D printed gingivoplasty guide, the procedure for gingivoplasty treatment becomes more efficient, precise, and easier to perform.

## Conclusions

To develop an effective treatment for GS and select an appropriate therapy, it is important to identify the underlying cause. In this case report, a patient with severe GS received orthodontic and gingivoplasty treatment, aided by digital tools such as 3D simulation, smile design, and 3D printed guides. The treatment yielded remarkable and satisfactory results, without the need for extensive surgery.
